# The influence of postural threat on strategy selection in a stepping-down paradigm

**DOI:** 10.1038/s41598-020-66352-8

**Published:** 2020-07-02

**Authors:** Nick Kluft, Sjoerd M. Bruijn, M. John Luu, Jaap H. van Dieën, Mark G. Carpenter, Mirjam Pijnappels

**Affiliations:** 1 Department of Human Movement Sciences, Vrije Universiteit Amsterdam, Amsterdam Movement Sciences, Amsterdam, The Netherlands; 2 Institute Brain and Behaviour Amsterdam, Amsterdam, The Netherlands; 3 Neural Control of Posture and Movement Laboratory, School of Kinesiology, The University of British Columbia, Vancouver, BC Canada

**Keywords:** Motor cortex, Ageing, Human behaviour

## Abstract

To walk safely in their environment, people need to select adequate movement strategies during gait. In situations that are perceived as more threatening, older adults adopt more cautious strategies. For individuals with excessive fear, selecting adequate strategies might be troubling. We investigated how a postural threat affects the selection of strategies within and between older adults by using a stepping-down paradigm. In twenty-four older adults we determined the height at which they switched in stepping-down strategies from a less demanding but more balance threatening heel landing to a more demanding yet safer toe landing. We expected that this switching height would be lower in the high (0.78 m elevation) compared to low threat (floor level) condition. Furthermore, we investigated if older adults, for which the postural threat evoked an increase in the perceived fear, presented a different stepping down strategy due to the postural threat. Our results indicated that the postural threat changed older adults’ strategies selection towards a more conservative toe landing. Hence, despite the additional effort, older adults prefer more cautious strategies during a postural threat. No effects of perceived fear on strategy selection between individuals were observed, potentially due to relatively small differences in fear among participants.

## Introduction

Older adults may have a perception of their physical abilities that is not in agreement with their actual physical abilities^1–3^. For example, Sakurai and colleagues^1^ showed that about one-third of older adults were not able to step over an obstacle, of which they believed that it would still be within their physical limits. In daily life, such mismatches may lead to dangerous situations. This disparity may cause selection of inappropriate motor behaviours^4^, which might be amplified when one is exposed to a challenging situation^5^. Yet, little is known whether and how these challenging situations affect the selection of movement strategies in gait.

Standing at an elevation can be challenging and such postural threat has been shown to affect the control of static motor tasks in both young and older adults^6^. This is shown through a tightened control of postural sway, i.e., reducing the centre of mass excursion, when standing on an elevated platform compared to standing on ground level^6–9^. In line with the more cautious control in standing, walking at a 0.60 m elevation caused older adults to walk slower and adopt a more conservative gait pattern, and these changes were more pronounced in older adults than in young adults^10–13^. Moreover, older adults seemed to select more cautious strategies, in terms of higher toe clearance, when crossing obstacles at elevation^12^. However, these studies did not constrain walking speed which might be problematic as walking slower is an effective strategy to reduce the risk of erroneous movement and may explain the observed changes in strategy selection.

To study the effect of postural threat on motor behaviour during gait in older adults, a stepping-down paradigm can be used^5^. When stepping down a small step height, this can be done by using a heel landing or toe landing, with preference towards the latter as the step height increases^14,15^. Participants presumably switch from heel landing to toe landing with increasing step height, to dissipate the kinetic energy generated by the height difference, despite the higher muscle activity associated with toe landing^16^. The change in strategy might be to control angular momentum as suggested by van Dieën and colleagues^16^. However, the results of Kluft and colleagues^5^ indicated that the dissipation of kinetic energy is the predominant factor as the linear and angular momentum were not different between toe and heel landings, when speed and step height were controlled for. As older adults can use both toe and heel landings, their strategy selection can be considered to reflect their willingness to trade-off increased effort (i.e., increased negative ankle muscle power) for decreased risk (i.e., increased dissipation of kinetic energy). We evaluated whether an increase in postural threat affected this trade-off in the strategy selection within older adults by using an elevation manipulation as described previously in studies on standing. Besides a shift in (sub)conciously selecting a more cautious stepping down strategy (i.e., by switching to a toe landing at smaller step heights), participants can alternatively also adopt safer behaviour within one strategy, for example with more cautious kinematic energy profiles.

Furthermore, with a postural threat, the consequence of falling due to balance loss increases. Therefore, a postural threat can evoke an increase in emotional responses such as fear and anxiety^6^. These responses and their influence on motor behaviour may differ between individuals. Davis and colleagues^8^ examined the association between height-related changes in balance control and fear between young adults. They found that fearful individuals at high threat leaned farther away from the platform edge and their centre of pressure featured larger amplitudes and higher frequencies than in less fearful peers. Similarly, during our stepping-down paradigm at elevation, older adults with higher perceived fear might respond stronger by choosing even more cautious behaviour than their less fearful peers.

In this study, our main aim was to study the effect of a postural threat on stepping down strategy selection *within* older adults. We hypothesised that a postural threat during stepping down would lead to the selection of more conservative toe landing strategies at lower step heights. This will be evident by a shift of the step height, at which individuals switch from heel to toe landing, towards lower values between a low to high threat condition. Furthermore, we hypothesised that participants are more cautious in stepping down in a high threat environment, even when they select a similar strategy than they did in the low threat condition. Hence, we predicted that the heel landing kinematic profiles at high threat would show adaptations reflecting a more cautious behaviour than at low threat. Our second aim was to explore whether *between*-subject difference in perceived fear relates to a change in stepping down strategy selection. We expected fearful older adults to select even more cautious strategies at lower step heights compared to their less fearful peers. A better understanding of how a postural threat and perceived fear affect movement strategy selection (within and between individuals) may help explain why some older adults fall while others with similar physical capacities do not.

This study is confirmatory and we pre-registered our hypotheses and research methods online (AsPredicted.org); which is available on the Open Science Framework (OSF) repository at (https://osf.io/fpzea/).

## Methods

### Participants

Older adults (aged 65 years and older) were recruited by word of mouth and we asked employees in residential homes, gyms, and other recreational facilities for seniors to spread advertisement posters. Potential participants were excluded from participation in this study if they had a mini-mental state examination score of 23 or lower, reported any neurological, musculoskeletal, or anxiety disorders, had uncorrectable visual impairment, suffered from a serious injury during the last 9 months, could only walk with a walking aid, were unable to walk independently for 10 consecutive minutes, or took medication which could affect gait stability. Participants gave informed written consent prior to any experimentation. The experiment was performed in accordance with the relevant guidelines and regulations and approved by the University of British Columbia Clinical Research Ethics Board (H18-01124).

### Efficacy, falls, and strength

Before the experiment, the participant’s fall concern was assessed, using the Falls Efficacy Scale International (FESi^17^). The self-reported number of falls in the last 6 months was recorded, and we administered the modified Gait Efficacy Scale (mGES^18,19^). For both hands, handgrip strength was determined in three assessments^20^ using a dynamometer (A5401-Digital Hand Grip Strength Dynamometer, Take, Niigata, Japan). Participants stood with their arms straight and parallel to their trunk, and the dynamometer was adjusted to fit their hand. To promote maximal effort, verbal encouragement was provided while participants squeezed the dynamometer^20^. The mean of the maximum value (from three attempts) recorded from each hand was used to determine the handgrip strength^20^.

### Stepping-down task

**Figure 1:**
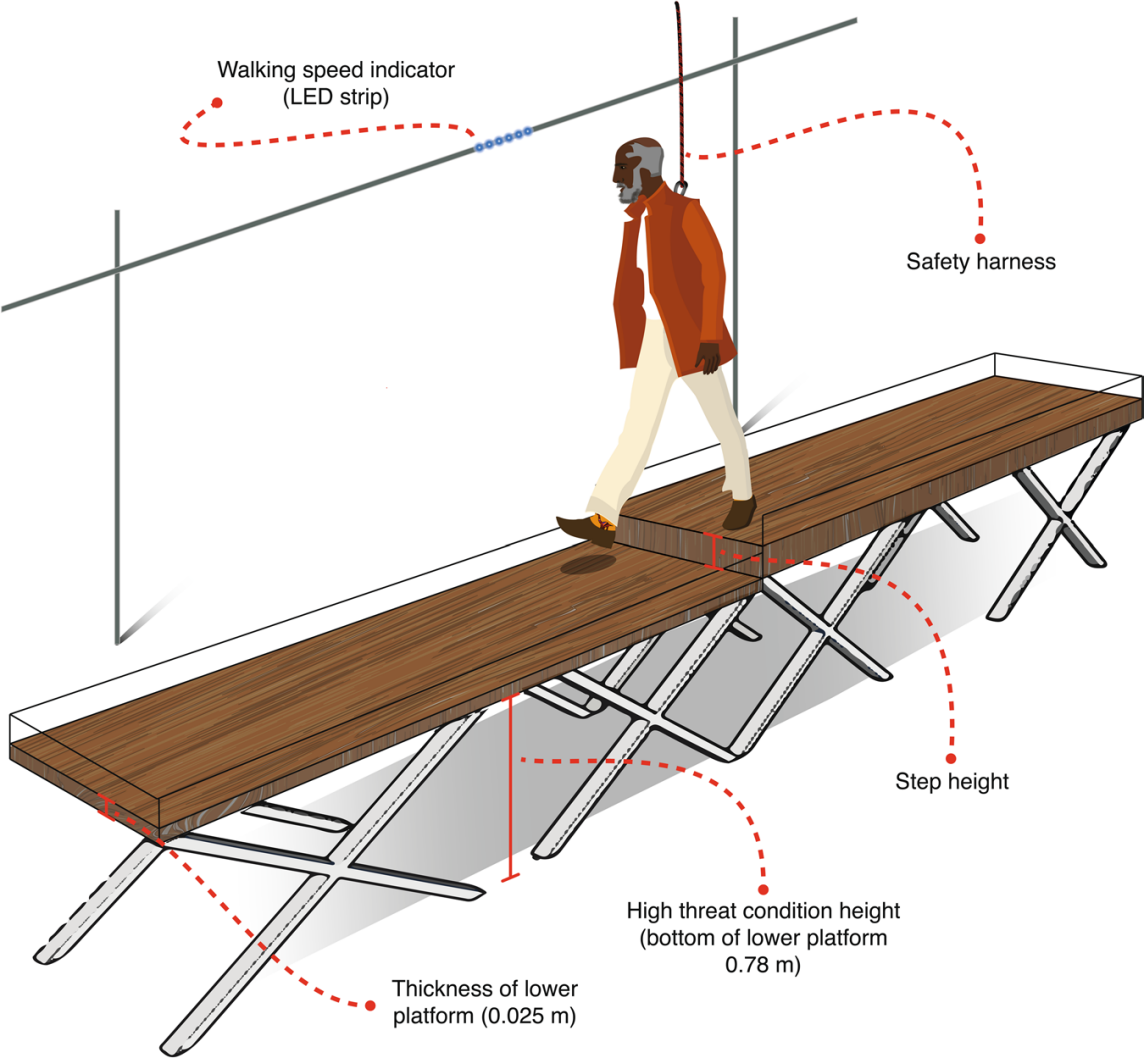
Experimental set-up of the postural threat condition. Participants were instructed to walk over a 4 m × 0.6 m walkway at an imposed walking speed indicated by LEDs that ran parallel to the walkway. Participants stepped down a height difference that was constructed in the middle of the walkway, and which was adjustable to multiple step-heights (ranging from 0.025 to 0.15 m). The walkway was secured by a small railing around the edge of the platform that assured foot placements within the platform boundaries. The low threat condition deviated solely from the high threat condition in terms of the height of the walkway (at floor level). Participants wore a safety harness during both experimental conditions.

Participants were asked to walk barefoot over a 2 by 0.6-m platform, stepping down on top of a second 2 by 0.6-m platform (Fig. 1). Participants performed the walking trials on bare feet, to avoid an effect of footwear on stepping-down strategy between individuals. In all trials, participants wore a safety harness. Walking speed was guided by a set of light-emitting diodes (LED) moving at a speed of 1.1 m per second alongside the platforms at eye height, and we instructed the participants to maintain a constant walking speed matching the LED speed. Walking speed affects the strategy selection (see van Dieën & Pijnappels^15^ for a detailed evaluation of this phenomenon). For the comparisons within and between individuals we wanted to exclude this effect from our results. Therefore, we controlled walking speed. Van Dieën & Pijnappels^15^ showed an approximately 50 percent chance of a heel landing (i.e., Pheel = Ptoe = 0.5) in older adults when walking at 1.1 m/s (4 km/h) with a 0.05 m step height. Thus, for assessment of the critical height, varying step height around the Pheel = Ptoe = 0.5 is most informative when the walking speed was set at 1.1 m/s. Participants were instructed to continue walking after stepping down, towards a highlighted area taped on the walking surface at the same speed. Then, we switched the heights of the two platforms, by sliding a movable top layer to the other platform, so the participant did not have to return to the starting position for the next trial. Participants waited shortly between trials, as it took only a few seconds for the experimenters to switch heights. Participants confronted up to six different step heights (0.025, 0.050, 0.075, 0.100, 0.125, and 0.150 m). The first evaluated step height was at 0.05 m. Participants performed six stepping-down trials per step height, after which this step height was changed, according to a protocol described previously^5^. The selected landing strategies (i.e., toe or heel landing) for each trial, within the range of step heights at which six heel landings and six toe landings occurred, was categorised by the experimenter by observation of the task in real-time. This complete stepping-down procedure was performed at ground level (i.e., low threat condition) and elevated 0.78 m above floor level (i.e., high threat condition). Previous literature observed gait and emotional changes at 0.60 m elevation^10–13^, therefore we were confident that an elevation of 0.78 m would be sufficient to induce a threat. Note, that the lower platform had a thickness of 0.025 m, meaning that participants walked at 0.025 m (low threat) and at 0.805 m (high threat), and that the top of the higher platform equals this elevation plus the step height. The order of the low- and high-threat conditions was randomised across participants. Prior to the protocol, participants completed an extensive set of familiarisation trials at ground level to minimise potential learning effects.

### Kinematics

A total of 7 LED infrared cluster markers was used to measure the movement of body segments and placed on the skin at the feet, calves, thighs, and pelvis. Two camera arrays (Optotrak, Northern Digital Inc., Waterloo, Ontario) were used to capture the 3D position of the cluster markers and sampled with a rate of 100 samples per second. Anatomical landmarks were identified and digitised using a pointer^21^.

### Perceived fear

We monitored the fear of falling related to each step-height block of six trials using a short questionnaire. The participant indicated how fearful they were by encircling a number, on a scale ranging from 1 (‘*I did not feel fearful at all*’) to 10 (‘*I felt completely fearful*’). If they felt that their fear should be placed between two numbers they were instructed to mark this on the line with a cross. The participants, for whom the ratings of perceived fear after the first block of six trials increased with threat condition, were classified as being fearful (i.e., fear group: Δfear > 0). As the perceived fear ratings are influenced by the time of exposure to the postural threat^22^, we only compared the perceived fear ratings after the first step down.

### Physiological arousal

Physiological arousal was assessed by recording electrodermal activity (EDA)^23^. Surface electrodes were placed at the base of the palm using common electrode adhesive and a hypoallergenic conducting gel. All areas immediately under the electrodes were cleaned to improve conductivity. The analog EDA signals were sampled at 1000 samples per second. The average of the EDA was determined for the entire time that the condition lasted, and expressed in μS. The difference in EDA (ΔEDA) between conditions (i.e., high-low) was computed and used for further analysis.

### Data analysis

A psychometric curve was fitted to the individual’s landing strategy data, the height at which both strategies were equally likely to occur was defined as the critical switching height (h_crit_)^5^. In the unlikely situation that all evaluated heights resulted in either toe or heel landings, h_crit_ was respectively set to 0.015 or 0.165 m.

A 3-D linked segment model was fitted to the kinematic data^24^, and gait events were detected automatically^25^, and visually verified afterward (i.e., we corrected the timing visually if the algorithm’s detection was off). Time series between the heel strike preceding the step-down, until the heel strike of the step following the step down were analysed. Missing data were interpolated using spherical linear interpolation^26,27^ thereby predicting segmental orientation, and when a complete cluster marker was not visible, one of the cluster markers was interpolated using a spline function.

Outcome measures were calculated from the resulting trajectories: (I) step length was determined by the distance between the feet during the double support phase in the forward direction, and (II) knee and ankle joint angles and angular velocities were calculated by a decomposition of the relative rotation matrices and their derivatives using Euler angles. Joint angles and angular velocities were time-normalised to the gait cycle.

Furthermore, investigation of the motor strategy requires constraining walking speed, as this could affect strategy selection^15^. To validate our experimental paradigm, walking speed was calculated as the mean of the first derivative of pelvis marker displacement along the travel path, which should not statistically differ between threat conditions.

### Sampling plan and statistical analysis

A Bayesian approach was used to test our alternative hypothesis that h_crit_ decreases in the high-threat condition, compared to the null hypothesis that there is no difference between the high- and low-threat conditions. To test this hypothesis, we used a Bayesian equivalent of a one-sided paired *t*-test, with a weakly informed prior distribution (i.e., a Cauchy distribution with an interquartile range of *r* = 1 centred around an effect size of 0^28^). We used a one-sided test, as a decrease in h_crit_ with an increase in threat level was expected. We monitored the Bayes factor during data collection until a threshold of meaningful evidence was reached^29–32^. We set this threshold to a Bayes factor (BF_10_) of 10, as this is generally considered as being ‘strong’ evidence in favour of *H*_1_ (or similarly a BF_10_ of 0.1 in favour of *H*_0_). First, twenty healthy older adults (age 65–85 years) were included, and this sample was extended until the BF_10_ exceeded the selected threshold, or a maximum of fifty participants was reached. In comparison with a frequentist approach, with a sample size of n = 50 subjects, an effect size of Cohen’s δ = 0.4 can be acquired with an 85% statistical power and a 5% Type I error rate. Finally, Bayesian statistical parametric testing (SPM^33–35^) was used to identify the kinematic differences between threat conditions. For this analysis, we solely used the kinematic trajectories in which a heel landing was observed. However, as the protocol was tailored per condition to the participant, there might be no overlap in strategy selection between conditions. To test whether older adults that reported an increased fear responded differently from their non-fearful peers, participants were categorised in groups, based on whether the postural threat affected their perceived fear of falling (fear group). A Bayesian repeated-measures analysis of variance (ANOVA) with threat-group interaction was used to evaluate whether fearful older adults respond differently to elevated walking – in terms of the strategy selection (h_crit_) – than their less concerned peers. For this analysis, JASP^36^ default priors were used (*r* = 0.5 for the fixed effects = 0.5 and *r* = 1 for the random effects) and Bayes factors were reported. A Bayesian linear regression was performed to explore whether the change in physiological arousal (ΔEDA) and the strategy selection in the low threat condition (h_crit_[low]) could explain the h_crit_ in the high threat condition (h_crit_[high]). For this analysis a default Jeffreys-Zellner-Siow prior with scaling factor 0.354 was used^36^. To evaluate the effectiveness of the postural threat and thus the validity of our manipulation, measures of perceived fear at the 0.05 m step height, physiological arousal averaged over the entire condition, and walking speed at 0.05 m step height were compared between threat conditions using Bayesian paired *t*-tests with one-sided Cauchy distributed priors (interquartile range *r* = 1, centred around an effect size of 0). We assumed our protocol to be effective when it increased the perceived fear and physiological arousal, and walking speed did not statistically differ under different threat conditions. To ensure that our findings did not mainly rely on the selected priors we assessed the robustness of the Bayes factor for all Bayesian tests^28,37^ (these results are reported in the OSF repository). Non-parametric alternatives were used, if the assumptions of a statistical test were violated, hence a Bayesian signed-rank test^38^ was applied for the tests that compared fear, anxiety, and walking speed across threat conditions. The Bayesian parametric hypothesis testing was performed in JASP^36^, and the SPM analysis (code adapted from https://osf.io/q5b72/ ^35^) and the non-parametric tests (code adapted from https://osf.io/gny35/ ^38^) were performed in R^39^.

### Deviations from the preregistration document

Three aspects that deviate from the registered document should be noted. First, we planned to determine the slope of the psychometric curve at h_crit_ to reflect the consistency of the strategy selection. In contrast to earlier studies^5,40^, we found an overall lower critical height in the present study. This led to a shift of the psychometric curve towards zero, which makes the slope of the curve less reliable as there were fewer data available to fit the lower end of the curve (due to the inability to evaluate stepping down at negative step heights). Hence, we omitted the consistency of strategy selection from further analysis. Second, for the handling of missing data, visual evaluation of the interpolated data demonstrated that the resulting trajectories were adequate, and there was no need to continue fitting linked-segment models, as suggested in the preregistered document. Third, for the between participant comparison, we planned to categorise fear group on the basis of the physiological arousal data. However, the grouping cutoffs appeared arbitrary. Instead we performed a linear regression, since no cutoffs are needed in a linear regression model and this analysis is analogous to the planned analysis.

## Results

**Figure 2:**
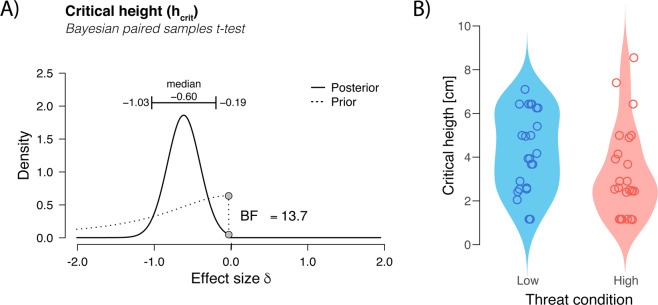
(**A**) Probability density distributions of effect size of h_crit_ between low and high threat conditions. The one-sided prior distribution is indicated by the dashed line. The posterior distribution, indicated by the solid line, equals the prior distribution updated by our observed data. The posterior density distribution indicates the likelihood of certain effect size (Cohen’s δ) on the horizontal axis. The 95% credible interval (95%CI) is indicated by the horizontal bar. Given the BF_10_, the alternative hypothesis that h_crit_ differs with threat condition is 13.7 times more likely than the null hypothesis that there is no difference between condition. (**B**) Violin plots of h_crit_, with jittered individual data points superimposed. Figures adjusted from JASP, jasp-stats.org.

**Figure 3:**
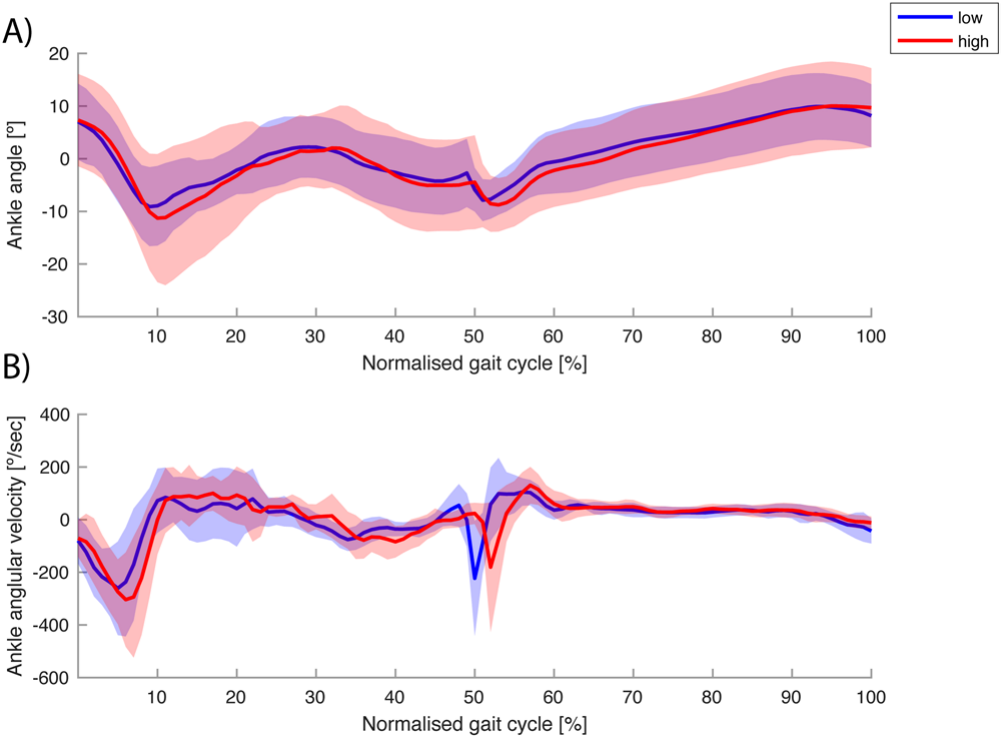
(**A**) Sagittal plane ankle joint angle (negative values indicate plantar flexion) and (**B**) angular velocity of the leading leg during stepping down under high (red) and low (blue) threat conditions. The shaded area displays ±1 standard deviation. Only heel landing profiles were selected for this comparison, and the average heel-landing trajectories within one condition and step height were averaged over participants. Time series are normalised from the heel strike before stepping down to the first step at the lower level (0–50%), and up to the next heel strike (51–100%).

**Figure 4:**
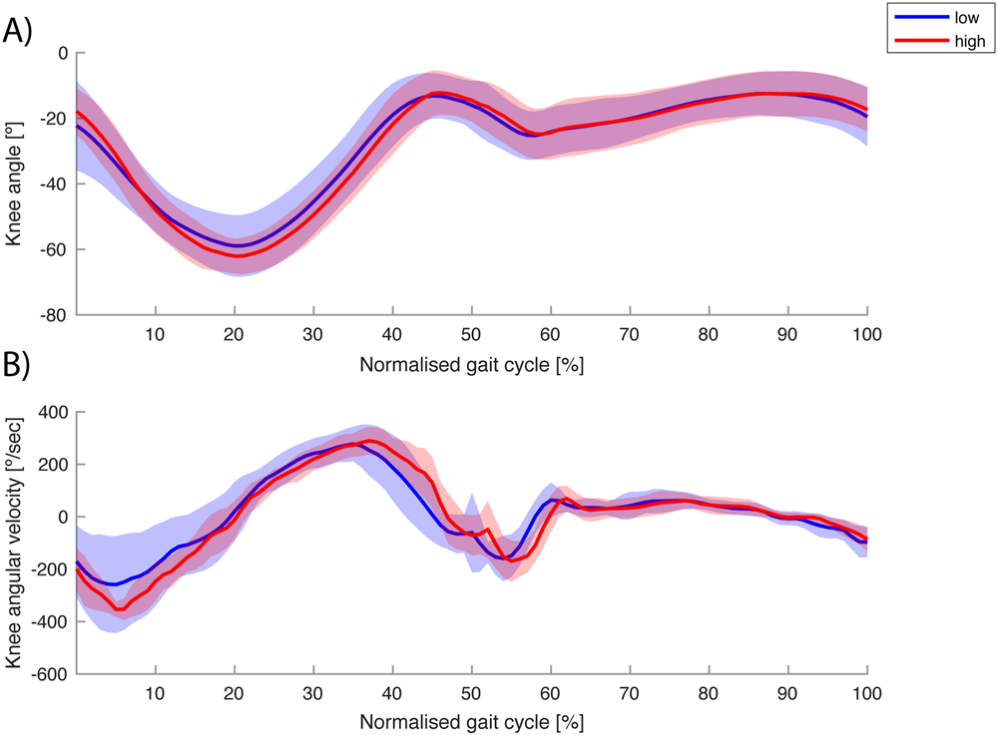
(**A**) Sagittal plane knee joint angle (negative values indicate knee flexion) and (**B**) angular velocity of the leading leg during stepping down under high (red) and low (blue) threat conditions. Only heel landing profiles were selected for this comparison, and the average heel-landing trajectories within one condition and step height were averaged over participants. The shaded area displays ±1 standard deviation. Time series are normalised from the heel strike before stepping down to the first step at the lower level (0–50%), and up to the next heel strike (51–100%).

Twenty-four healthy older adults were measured (see Table 1 for participants’ descriptives), of which 14 participants started in the low threat condition and 10 participants started in the high threat condition. When exposed to the high-threat condition, participants lowered h_crit_ (mean h_crit_ low = 0.043 ± 0.018 m, mean h_crit_ high = 0.033 ± 0.020 m, Cohen’s δ = −0.59 with 95%CI [−1.03, −0.19], and BF_10_ = 13.7). Figure 2 presents h_crit_ under low and high-threat conditions and the probability density distributions of the effect size δ. Furthermore, step length during the step down was slightly longer in the high-threat condition compared to the low-threat condition (mean low = 0.54 ± 0.06 m, mean high = 0.57 ± 0.06 m, Cohen’s δ = 0.59, BF_10_ = 5.0). However, this finding was probably due to the change in landing strategy, resulting in relatively more toe landings, as this effect did not remain when only heel landings were compared (mean low = 0.58 ± 0.06 m, mean high = 0.57 ± 0.06 m, Cohen’s δ = −0.15, BF_10_ = 0.3). Note that the evaluated step heights and their order of appearance depended on participant’s behaviour and could vary between conditions. Therefore, the analysis of comparing heel-landing strategies was only possible for a subset of participants (n = 13), who selected heel landings in both conditions at comparable step heights. Normalised ankle joint and knee joint angles and velocities are depicted in Figs. 3 and 4. The SPM analyses of the heel-landing profiles showed that knee and ankle joint angles and angular velocities did not differ between conditions. For the between-subject comparison, we found in seven participants the postural threat affected their self-reported fear. This fear group had a median increase of 1 (IQR = 0.75) on the perceived-fear scale. Repeated measures Bayesian ANOVA revealed that the (threat × group) interaction effect model was not preferred over the main effect models (fear group model: ${\eta }_{p}^{2} < 0.001$, BF_10_ = 0.41). Hence, the data are uninformative and provide no evidence that could support our hypothesis that fearful individuals would or would not respond differently – in terms of h_crit_ – on threat condition than their peers. Note, that despite the between-group sample size differences, the ANOVA’s assumptions were not violated. The change in physiological arousal between conditions (ΔEDA) in the Bayesian linear regression did not improve the fit of the model (*R*^*2*^ = 0.45, BF_10_ = 0.33). Hence, the change in physiological arousal did not explain differences in h_crit_ between the low and high-threat conditions. Walking in the high-threat condition increased physiological arousal (mean low = 16.8 ± 10.0 μS, mean high = 18.6 ± 8.9 μS, Cohen’s δ = 0.85, and BF_10_ = 212.2). Perceived fear was marginally affected by threat condition (median low = 1 IQR 1 point, median high = 1 IQR 0.5 point, and BF_10_ = 0.7). Walking speed was slightly lower than indicated by the set of LEDs in the high-threat condition (mean low = 1.08 ± 0.13 m/s, mean high = 1.04 ± 0.14 m/s, Cohen’s δ = −0.49, and BF_10_ = 4.6).

**Table 1:** Participant descriptives, response, and explanatory variables.

*Descriptive variables* (*N* = *24 older adults*)				
Age	73.8	(4.9)	M(SD)	years
Females	14	(58.3%)	n(%)	persons
Fallers	8	(33.3%)	n(%)	persons
Fall history	0	[1]	Mdn[IQR]	falls
MMSE	29	[1]	Mdn[IQR]	points
Body weight	66	[26.7]	Mdn[IQR[	kg
Body height	1.66	(0.1)	M(SD)	m
Grip strength	23.7	[12.6]	Mdn[IQR]	kg
FESi	19	[6.5]	Mdn[IQR]	points
mGES	97.5	[7.5]	Mdn[IQR]	points
	*Low threat*	*High threat*	(*High-Low*)	
Within variables	M ± SD	M ± SD	Cohen’s δ	BF_10_
Physiological arousal [S]	16.8 ± 10.0	18.6 ± 8.9	0.85	212.2
h_crit_ [m]	0.043 ± 0.018	0.033 ± 0.020	−0.59	13.7
Walking speed [m/s]	1.07 ± 0.13	1.03 ± 0.14	−0.48	4.4
Step length* [m]	0.58 ± 0.06	0.57 ± 0.06	−0.16	0.3
	Mdn [IQR]	Mdn [IQR]		BF_10_
Perceived fear [points]	1[0]	1[0.5]		0.7

When the variable was normally distributed the mean with standard deviation M(SD) was reported, otherwise, the median with interquartile range Mdn[IQR] was reported. Solely for count data, the number and percentage n(%) of the total sample size was reported.

*Considering only stepping downs that considered heel landing strategies.

## Discussion

Our results confirmed our initial hypothesis that older adults adjusted their stepping-down behaviour when under threatening conditions. This is in line with our expectation that postural threat causes older adults to behave more cautiously, and therefore switch to toe landings at lower heights. Changing stepping-down behaviour under threat may contribute to an increased sense of safety, and may be protective in daily-life situations, despite the increased effort in terms of muscle force production^16^. Whereas a postural threat due to elevation increases the potential consequence of inadequate strategy selection, do other circumstances, such as blurred vision, fatigue or weather conditions increase the probability of an inadequate execution of the selected strategy and may therefore also lead to the selection of more cautious or more robust movement strategies. Moreover, the increased arousal that our participants experienced at elevation may replicate that experienced by individuals who suffer from increased fear of falling during daily-life activities. This implies that older adults with excessive concern of falling may select overly cautious movement strategies during daily-life tasks.

Previous work on stepping-down behaviour suggested that strategies are selected based on a trade-off between safety and efficiency^15,41^. This study adds evidence for this trade-off as older adults preferred the safer strategy when walking on elevation. Between individuals, we found no evidence for an interaction effect of threat with perceived fear on the change in strategy selection of stepping down across threat conditions. The absence of such an interaction effect might be due to the threat of our experimental manipulation; the variability on the self-reported perceived fear was only marginal, which might be due to our modest platform elevation of 0.78 m. For comparison, Davis and colleagues^8^ evaluated standing at heights of 0.8 m, 1.6 m and 3.2 m and found robust fear responses only at the 3.2 m height. Even though walking is more challenging than standing at an elevated height, it is recommended for future research to study gait at higher elevations (>0.78 m) to distinguish how different psychological conditions contribute to altered strategy selection in older adults. Delbaere and colleagues^13^ found in their study, in which they compared walking at floor level and at 0.6 m elevation, mean values of 16.29 μS and 16.32 μS respectively (which is comparable with our findings of 16.8 μS and 18.6 μS). However, like comparing EMG between individuals, comparing EDA values between individuals is often not informative. Values largely depend on room temperature, skin composition, electrode placement, and the type of electrode gel. However, the within participant comparison is valid as these factors are not altered.

We evaluated the knee and ankle joint kinematics to detect differences in control between threat conditions within either toe landing or heel landing strategies. The mechanical challenge of stepping down is to keep control of the angular momentum and kinetic energy induced by the height difference^5,16^. Even though some participants still selected heel-landing strategies when walking under high threat, the threat-related adjustment could have been governed by adaptations, reflected in different kinematic trajectories. However, we did not observe any difference in the joint kinematics between heel landings across conditions. Another effective strategy is to adjust the foot placement to increase the step length^42^. We had expected the step length to increase with the postural threat to allow more reduction of angular momentum at landing; however, our data do not provide the evidence to support this hypothesis. These findings confirm that older adults adjusted to the postural threat by making a discrete switch in stepping-down strategy.

The small effect sizes of the ANOVA’s threat × group interaction and linear regression suggest that the postural threat does not alter the stepping-down strategy of the more aroused or fearful older adult relative to their peers. In this study, we assumed that the strategy selection is the variable through which the safety during stepping down is controlled. This seems to be an acceptable assumption, given that the postural threat decreased h_crit_. So, why is the strategy selection not different between our fear groups? This is probably due to the relatively small effect of the postural threat on the emotional state of the participant. Although we measured an increased physiological arousal at elevation, there was only a slight increase in reported fear in a few participants. Hence, the instrument used to measure the perceived fear was not sensitive enough to indicate the small differences caused by our manipulation.

This study aimed to understand how a postural threat, induced by a height manipulation, affects movement strategy selection in older adults. The validity of our threat manipulation was assessed by evaluation of the perceived fear, physiological arousal, and walking speed. The increase in physiological arousal suggests the manipulation imposed a postural threat and provoked a physiological arousal response^6^. However, the ratings of the perceived fear, were unaffected by the imposed threat, suggesting that the elevation in the high threat condition was not sufficient to induce a significant change in the perceived fear. Moreover, the seven participants for whom the perceived fear increased had only a slight change in score, which was not practically relevant.

Different from previous studies described in the introduction^10–13^, we imposed the walking speed using a LED strip. Despite the imposed walking speed, the postural threat drove participants to walk slightly slower compared to walking at ground level. A previous study on strategy selection during stepping down showed that the strategy selection is a function of walking speed^15^. As walking speed differed between threat conditions, this may have affected switching heights. However, the observed difference in walking speed was relatively small (<0.05 m/s), implying that the magnitude of this difference is not sufficient to explain changes in stepping-down strategy. Interestingly, no differences in step length between conditions were observed; therefore, we believe that the previously reported differences in step length can be (mainly) explained by the major change in walking speed over conditions. Future research studying effects of a postural threat on human gait, should consider controlling for walking speed.

This study had some limitations to be considered. In this experiment, participants walked barefoot, which most likely reduced the probability of stepping down with a heel-landing strategy. Indeed, we observed lower h_crit_^5,40^, compared to earlier work on stepping-down strategies in which participants wore footwear. We controlled for walking speed with a LED strip, but this required participants to adapt their walking speed to that of a moving light beam. Participants could have been distracted by the light beam. However, we did not expect great deviations due to such distraction, as participants were well acquainted with the walking speed and the light beam (i.e., they performed an extensive familiarisation trial). Although, we tried to constrain walking speed, our results showed a minor difference in walking speed between conditions, which might have affected the results as suggested above. Our participants were required to be able to walk for at least 10 consecutive minutes independently. This may have caused a selection bias as solely fit older adults (mean grip strength = 23.7 kg) with a low fear of falling experienced during daily life activities were recruited (median FES-i = 19^43^ established cutoffs of 16–19 for low concern and 20–27 for moderate concern). These individuals may have been less susceptible to the postural threat as they have higher confidence in their gait ability. Furthermore, participants wore a safety harness, which likely reduced the response – in terms of strategy selection, joint kinematics, perceived fear, and physiological arousal – to the postural threat.

In accordance with the initial and preregistered hypothesis, our findings indicate that older adults change their motor behaviour, in terms of strategy selection in a stepping-down paradigm, due to a postural threat imposed by walking at elevation. Despite the additional effort of the selected landing strategy, older adults adjusted their motor strategy selection in response to the imposed threat towards using a toe landing.

## Data Availability

The datasets analysed for this study and all figures can be found in an Open Science Framework repository (https://osf.io/eu2d6/).
